# Multi-Omics Reveal the Immunological Role and the Theragnostic Value of miR-216a/GDF15 Axis in Human Colon Adenocarcinoma

**DOI:** 10.3390/ijms222413636

**Published:** 2021-12-20

**Authors:** Chun-Bin Tung, Chia-Ying Li, Hung-Yu Lin

**Affiliations:** 1 Department of Emergency Medicine, Show Chwan Memorial Hospital, Changhua 500, Taiwan; aclsacls@yahoo.com.tw; 2 Department of Surgery, Show Chwan Memorial Hospital, Changhua 500, Taiwan; 3 Graduate Institute of Biomedical Engineering, National Chung Hsing University, Taichung 402, Taiwan; 4 Research Assistant Center, Show Chwan Memorial Hospital, Changhua 500, Taiwan

**Keywords:** colon adenocarcinoma, miR-216a, growth differentiation factor 15, diagnostic biomarker, prognostic biomarker, immunomodulation, immune infiltration, precision treatment, Gemcitabine

## Abstract

Colon adenocarcinoma (COAD) is the most common type of gastrointestinal cancer and is still the third leading cause of cancer-related mortality worldwide. Accurate screening tools for early diagnosis and prediction of prognosis and precision treatment strategies are urgently required to accommodate the unmet medical needs of COAD management. We herein aimed to explore the significance of the microRNA (miR)-216a/growth differentiation factor 15 (*GDF15*) axis in terms of clinical value, tumor immunity, and potential mechanisms in COAD by using multi-omic analysis. The gene expression levels of miR-216a and *GDF15* showed an increase in the COAD group compared to those of the normal group. The expression of miR-216a presented a negative correlation with *GDF15* in COAD tumor tissue. The use of an in vitro luciferase reporter assay and bioinformatic prediction revealed that miR-216a-3p acted toward translational inhibition on *GDF15* by targeting its 3′untranslated region (UTR) site. High miR-216a expression was associated with decreased overall survival (OS), while the high expression of *GDF15* was associated with increased OS. Enriched type 1 T-helper (Th1), enriched regulatory T (Treg), enriched eosinophils, and decreased nature killer T-cells (NKTs) in COAD tumor tissue may play counteracting factors on the tumor-regulatory effects of miR-216a and *GDF15*. In addition, high *GDF15* expression had associations with suppressed immunoinhibitory genes and negative correlations with the infiltration of macrophages and endothelial cells. The enrichment analysis revealed that *GDF15* and its co-expression network may be implicated in mitochondrial organization, apoptosis signaling, and endoplasmic reticulum (ER) stress response. The Genomics of Drug Sensitivity in Cancer (GDSC) and Cancer Therapeutics Response Portal (CTRP) analysis identified that Gemcitabine acted as a precision treatment for COAD when *GDF15* expression was low. This study supports the miR-216a/*GDF15* axis as a diagnostic/prognostic panel for COAD, identifies Th1, Treg, eosinophils, and NKTs as counteracting factors, indicates potential relationships underlying immunomodulation, mitochondrial organization, apoptotic signaling, and ER stress and unveil Gemcitabine as a potential drug for the development of treatment strategy when combined with targeting *GDF15*.

## 1. Introduction

Colon adenocarcinoma (COAD) is the most frequently diagnosed histological subtype of colorectal cancer (CRC), which is the third most common type of malignant tumor, and in 2020, it ranked second in terms of cancer-related deaths globally [1]. In recent decades, considerable progress has been made regarding early diagnosis and multidisciplinary management strategies; however, the invasion, migration, metastasis, and recurrence of COAD have been challenges for improving long-term survival rates, preventing the 5-year survival rate from exceeding 30% [2,3]. Therefore, the development of highly accurate screening tools for the early diagnosis and prediction of prognosis, as well as precision treatment strategies, is urgently required to accommodate the unmet medical needs of COAD management.

Growth differentiation factor 15 (GDF15) is a well-known endocrine-acting mitokine, which is induced and secreted in response to mitochondrial stress [4]. GDF15 belongs to the transforming growth factor β (TGF-β) superfamily and thus presents the characteristic structure of the TGF-β superfamily [5,6]. The precursor protein of GDF15 contains 308 amino acids. After furin protease cutting, it becomes a mature protein and is subsequently secreted from the cell. GDF15 exerts autocrine and paracrine functions both on the cell, and it is secreted from the surrounding cells [5,6]. In a normally functioning human body, the expression of GDF15 exhibits tissue specificity, primarily distributed in the prostate, kidney, and pancreas [7], although its expression is increased at the kidney and liver lesions of several cancers [7,8]. However, the role of GDF15 in COAD remains controversial. For example, elevated GDF15 levels as a poor prognostic marker and tumor promoter have been noted [9,10,11], whereas its tumor-suppressive role has also been revealed [12].

microRNAs (miRs) are small non-coding RNAs with approximately 22 nucleotides, which play a regulatory role in the transcriptional control mechanisms for the maintenance of metabolic homeostasis. miRs are transcribed by RNA polymerase II or III in the nucleus to generate primary miRs (pri-miRs), which are then processed to become precursor miRNAs (pre-miRs) [13]. The miRs subsequently bind to the 3′untranslated region (3′UTR) of target mRNA(s), which can affect mRNA degradation or translational repression [14]. Despite displaying such repressive activity, it has been noted that some miRs act to enhance gene expression [15]. In addition, mechanisms including miRNAs binding to 5′UTR or the coding sequence of mRNA [16], toll-like receptors [17,18], or mitochondrial transcripts [19] have recently been reported. While the tumor-suppressive role of miR-216a in COAD has been shown [20,21], a more recent study reported miR-216a presents a pro-COAD effect [22]. Thus, more studies are warranted to clarify this debatable issue.

The purpose of this study was to explore the significance of the miR-216a/*GDF15* axis in terms of clinical value, tumor immunity, and potential mechanisms in COAD. We first explored the gene expression profiles and the association of miR-216a and GDF15 in human samples of COAD and confirmed the miR-216a/GDF15 interaction at the transcriptional level. Second, we investigated the prognostic value of miR-216a and *GDF15* and found the counteracting factors based on immune cell content. Third, we analyzed the correlation between GDF15 and immunoinhibitory markers and immune infiltration. Next, we employed a gene enrichment approach to reveal the molecular mechanisms and biological pathways of GDF15 in COAD. Finally, we combined Genomics of Drug Sensitivity in Cancer (GDSC) and Cancer Therapeutics Response Portal (CTRP) analyses to identify that Gemcitabine acts to simulate the GDF15 gene profile and is a potential candidate for the treatment of COAD. Our results may support the use of miR-216a and GDF15 as diagnostic/prognostic biomarkers for COAD, further clarify the molecular basis underlying immunomodulation, mitochondrial organization, and apoptotic signaling and reveal Gemcitabine as a potential precision treatment for COAD.

## 2. Materials and Methods

### 2.1. Multi-Omic Analysis

The coding gene expression profiles across various cancer types were accessed from UALCAN (http://ualcan.path.uab.edu/; accessed on 25 October 2021) [23] and GEPIA2 (http://gepia2.cancer-pku.cn/#index; accessed on 25 October 2021) [24]. The non-coding gene profiles were obtained from The Encyclopedia of RNA Interactomes (ENCORI; starBase v3.0) web server, an open-source platform for pan-cancer analysis on the miRNA-ncRNA, miRNA-mRNA, ncRNA-RNA, RNA-RNA, RBP-ncRNA, and RBP-mRNA interactions from CLIP-seq, degradome-seq, and RNA-RNA interactome data [25]. We utilized the Human Protein Atlas (http://www.proteinatlas.org; accessed on 25 October 2021), a Swedish-based program initiated in 2003 to map human proteins in cells, tissues and organs, to determine the immunohistochemical (IHC) staining signal of GDF15 expression [26,27,28]. The validation test data for the differential expression of miR-216a were assessed with the OncoMir Cancer Database (OMCD, a repository enabling systemic comparative genome analysis of miR expression sequencing data derived from over 10,000 cancer patients with associated clinical information and organ-specific controls present in TCGA (https://www.oncomir.umn.edu/omcd/; accessed on 25 October 2021) [29] and GDS2609 of the Gene Expression Omnibus (GEO, a database repository of high throughput gene expression data and hybridization arrays, chips, and microarrays). The prediction of the direct binding between miR-216a and *GDF15* 3′UTR was conducted by TargetScanHuman v8.0 (http://www.targetscan.org/vert_80/; accessed on 25 October 2021). The mutation landscape was retrieved in cBioPortal (https://www.cbioportal.org/; accessed on 25 October 2021), which is a web platform of gene-based data exploration. The Kaplan–Meier analysis based on gene expression levels was conducted in the Kaplan–Meier plotter web server (available online: https://kmplot.com/analysis/; accessed on 25 October 2021), which includes data sources from the GEO, the European Genome-phenome Archive (EGA), and TCGA to enable the assessment of the effect of 54,000 genes, including mRNA, miR, and protein on survival in 21 cancer types [30]. In addition, the OncoLnc web server permits users to link TCGA survival data to mRNA and miRNA expression levels (http://www.oncolnc.org/; accessed on 25 October 2021). The construction of protein–protein interaction (PPI) and functional enrichment analysis were conducted by using GeneMANIA v3.6.0 (https://genemania.org/; accessed on 25 October 2021) and STRING v11.5 (https://string-db.org/; accessed on 25 October 2021). The enrichment analysis integrating gene ontology sources including Gene Ontology (GO), Kyoto Encyclopedia of Genes and Genomes (KEGG), Reactome gene sets, and canonical pathways was conducted by Metascape algorithm [31] (https://metascape.org/gp/index.html#/main/step1; accessed on 25 October 2021). The drug sensitivity profiling based on GDF15 expression was examined by the CRISPR-screen data repository of GDSC algorithm in Q-omics v0.95 (accessed on 1 November 2021) [32] and by CTRP-based algorithm in GSCALite (accessed on 1 November 2021) [33]. The expression of each gene in the gene set based on GSCALite was performed by Spearman correlation analysis with the CTRP drug sensitivity (half maximal inhibitory concentration, IC_50_) data. The positive correlation means that the low expression of the gene is sensitive to the drug, and vice versa. The filtering criteria for multi-omic analysis were stated in Supplementary Materials.

### 2.2. In Vitro Cell Experiment

**Figure 1:**
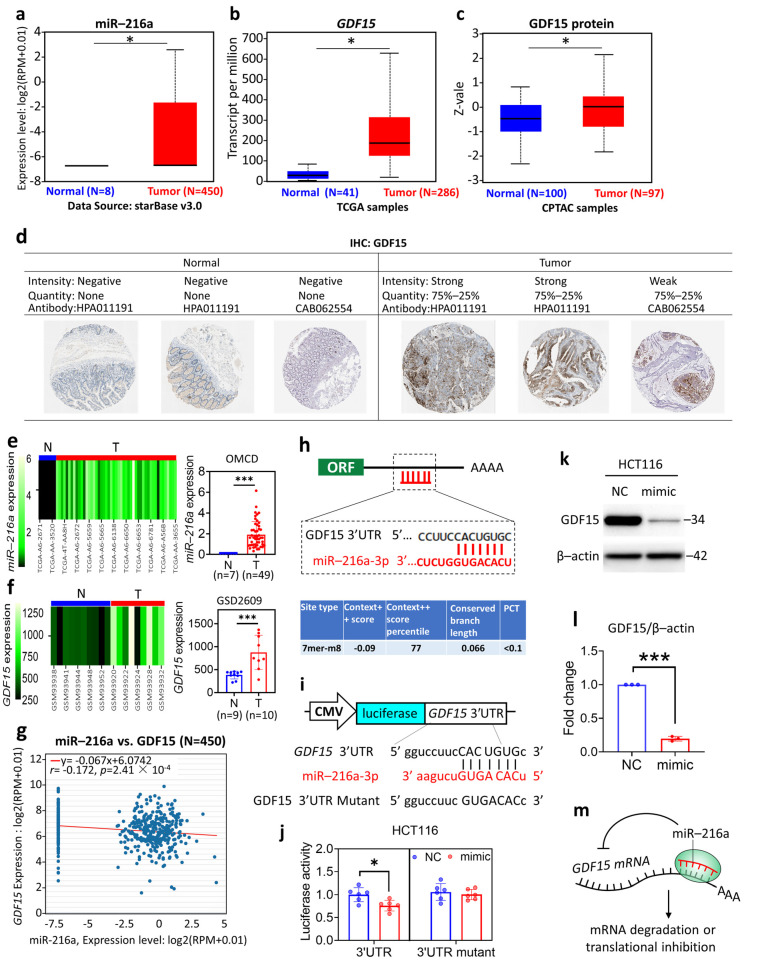
Differential expression analysis and the molecular interaction of microRNA (miR)-216a and growth differentiation factor 15 (*GDF15*). The gene expression levels of miR-216a (**a**) and GDF15 (**b**) in normal and colon adenocarcinoma (COAD) tumor tissue. The protein expression levels (**c**) and the immunohistochemistry staining (**d**) of GDF15. (**e**) Heatmap and histogram of the expression levels in normal and COAD tumor tissue retrieved from OMCD (N, normal; T, tumor). (**f**) Heatmap and histogram of *GDF15* expression levels in COAD tumor tissue retrieved from GSD2609 of the GEO database (N, normal; T, tumor). (**g**) Pearson’s correlation of miR-216a and *GDF15* gene expression levels retrieved from The Encyclopedia of RNA Interactomes (ENCORI). (**h**) Target scan human-based bioinformatics prediction of the complementary relationship between miR-216a-3p and *GDF15* 3′untranslated region (UTR). (**i**) Graphic illustration of the luciferase reporter system for the detection of miR-216a-3p/*GDF15* 3′UTR interaction. (**j**) miR-216a-3p-mediated suppression of luciferase signal in HCT116 cells. Cells were treated with miR negative control (NC) or miR-216a-3p (mimic) for 24 h. Six independent experiments for each group were conducted. (**k**) Representative Western blotting image of the expression levels of GDF15 and β-actin. (**l**) Quantification results of GDF15 normalized to β-actin. (**m**) The illustration of the inhibitory activity of miR-216a on *GDF15* by direct binding to its mRNA 3′UTR. * *p* < 0.05, *** *p* < 0.001 between groups.

HEK293, HEK293T, and HCT116 cell lines were cultured at 5% CO_2_ and 37 °C in DMEM (Thermo Scientific, San Diego, CA, USA) supplemented 10% FBS (Thermo Scientific), penicillin (100 U/mL; Thermo Scientific), and streptomycin (100 µg/mL; Thermo Scientific). The *GDF15* 3′UTR or *GDF15* 3′UTR mutant sequences were cloned into the multiple cloning site of pMIR-REPORTERTM plasmid after cytomegalovirus (CMV) promoter-driven luciferase (Figure 1i). To observe luciferase signals, the reporter plasmid was subsequently introduced into the HEK-293 cells, which were then treated with miR negative control sequence (HMC0003; Sigma-Aldrich^®^, St. Louis, MO, USA), miR-216a-3p mimic (HMI1979; Sigma-Aldrich^®^), or no treatment (NT). The HEK-293 cells were seeded at 1.5 × 10^6^ cells in a 100 mm dish for 18 h. Five micrograms of reporter plasmids with *GDF15* 3′UTR or *GDF15* 3′UTR mutant were then individually transfected into the HEK-293 cells using Lipofectamine™ 2000 Transfection Reagent (11668030; Thermo FisherScientific, Rockford, IL, USA) for 16 h. The cells were then trypsinized and seeded at 7.5 × 10^5^ cells in a 60 mm dish with a fresh growth medium. Subsequently, 20 nM miR-216a-3p mimic, miRs negative control, or NT were transfected into cells with RNAiMAX transfection reagent (13778-150; Invitrogen, Carlsbad, CA, USA) for 24 h. The cells were then lysed to detect luciferase signals with the Neolite Reporter Gene Assay System (PerkinElmer, Waltham, MA, USA). The procedure of detecting luciferase signals was followed according to the standard protocol of the manufacturer of the Neolite Reporter Gene Assay System. For Western blotting, 30 μg proteins were separated in SDS-PAGE gels and were transferred onto 0.45-µm PVDF membranes (Millipore, Burlington, MA, USA) in a Trans-Blot^®^ SD Semi-Dry Transfer Cell (Bio-Rad). The membrane was blocked in 5% non-fat milk powder/PBST (1X PBS, 0.1% Tween-20 (Sigma-Aldrich, St. Louis, MO, USA) and incubated overnight at 4 °C with a blocking buffer containing primary antibodies. The membrane was washed and then incubated for 1 h with 5% non-fat milk powder and a PBS-T-containing secondary antibody. The signals were developed using the Ultra ECL-HRP Substrate (#TU-ECL02, TOOLS) using X-ray films. Primary antibodies were purchased from Santa Cruz Biotechnology (anti-GDF15 (sc-377195) and anti-β-actin (sc-8432)), and the secondary antibody was purchased from Jackson ImmunoResearch (111-035-003).

### 2.3. Immunological Databases

The correlation between *GDF15* and the immunoinhibitory genes in COAD was analyzed by TISIDB, which is an integrated repository web portal for tumor-immune system interactions [34]. The association between GDF15 expression and immune cell infiltration in COAD was determined using The Tumor Immunology Estimation Resource version 2.0 (TIMER 2.0), which is an online tool for systematically assessing the expression of gene sets associated with infiltrating immune cells in TCGA datasets.

### 2.4. Construction of PPI Network and Enriched Analysis

The significantly co-expressed genes of GDF15 were determined by the correlation repository of UALCAN. The genes displaying prognostic significance in patients with COAD were selected for PPI analysis using GeneMANIA and STRING. GeneMANIA is an open website for building PPI networks and demonstrating gene function [35,36]. By applying bioinformatics methodologies, this website analyzes genes or gene lists, which includes data regarding physical interaction, co-expression, and co-location, as well as enrichment and predictive analyses. To determine PPI, we utilized STRING, which is an online data-mining platform and includes data regarding physical and functional interplays [37].

### 2.5. Statistical Analysis

All values are expressed as mean ± standard error (SE). Quantitative data were analyzed using unpaired t-test for two-group comparison or one-way analysis of variance (ANOVA) for three- or more-group comparison when appropriate. Bonferroni test was used for post-hoc analysis. Two-sided *p*-values less than 0.05 were considered to be statistically significant.

## 3. Results

### 3.1. Differential Expression Profile and the Molecular Interaction of miR-216a and GDF15

The gene expression levels of miR-216a and *GDF15* showed an increase in the COAD group compared to in the normal group (Figure 1a,b). The protein expression levels of GDF15 presented a similar profile (Figure 1c). In addition, the IHC-determined GDF15 protein expression presented a higher quantity of GDF15 staining signals in COAD than in normal tissue (Figure 1d), in line with the aforementioned findings. The miR-216a expression levels retrieved from the OncoMir Cancer Database (OMCD) (Figure 1e) and the *GDF15* accessed from GDS2609 of GEO (Figure 1f) database confirmed the findings. We noted that the expression of miR-216a presented a negative correlation with GDF15 in COAD tumor tissue (Figure 1g). We thus investigated whether miR-216a acts to suppress *GDF15* at the transcriptional level. The TargetScanHuman survey predicted that miR-216a-3p has a complementary relationship with *GDF15* at its 3′UTR site (Figure 1h). To verify this, we employed a luciferase reporter assay, in which a CMV-driven plasmid was inserted with a fusion sequence of luciferase with *GDF15* 3′UTR or *GDF15* 3′UTR mutant (Figure 1i). Then, the luciferase reporter plasmid was transfected into the HCT116 colorectal carcinoma cell line. The luciferase activity was decreased by the presence of miR-216a-3p mimic, while unaltered in cells harboring the *GDF15* 3′UTR mutant (Figure 1j), suggesting the direct binding of miR-216a to *GDF15* 3′UTR. Similar results were seen in HEK-293 and HEK-293T cells (Supplementary Figure S1). The Western blotting results confirmed that miR-216a-3p mimic suppressed the protein expression level of GDF15 (Figure 1k,l). Collectively, these results indicated that miR-216a and *GDF15* featured the diagnostic value for COAD and that miR-216a targeted 3′UTR of *GDF15* to suppress its expression.

### 3.2. Prognostic Value of miR-216a and GDF15

**Figure 2:**
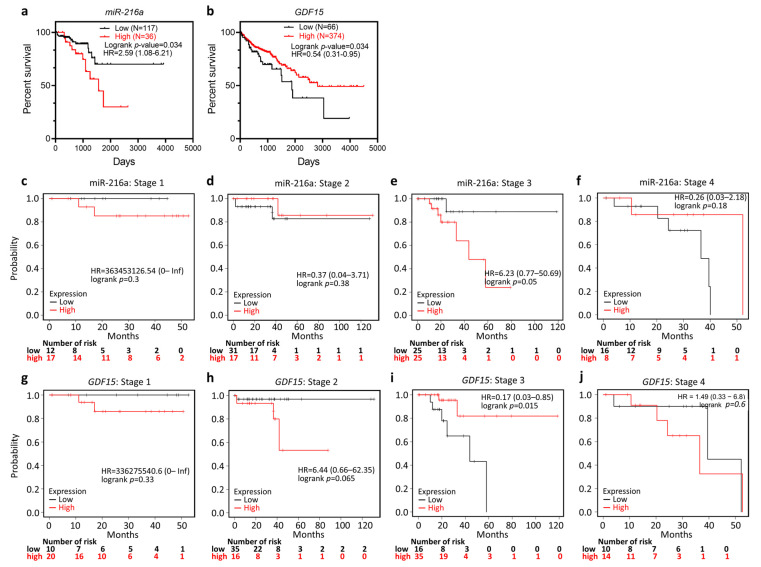
Prognostic value of miR-216a and GDF15. The probability of the overall survival (OS) in COAD patients based on low vs. high gene expression of miR-216a (**a**) and GDF15 (**b**) based on the Kaplan–Meier survival analysis. Patients at stages 1 (**c**,**g**), 2 (**d**,**h**), 3 (**e**,**i**), and 4 (**f**,**j**) were individually analyzed.

The correlation between the expression of miR-216a and *GDF15* and the corresponding clinical follow-up information was analyzed by Kaplan–Meier curves and the logrank test. High miR-216a expression was found to be associated with decreased overall survival (OS; Figure 2a), while the high expression of *GDF15* was associated with increased OS (Figure 2b). Specifically, miR-216a and *GDF15* served as independent prognostic factors in COAD patients with stage 3, but not in stages 1, 2, and 4 (Figure 2c–j). These results indicated the prognostic significance of the miR-216a/GDF15 axis, especially at the late stage.

### 3.3. Prognostic Value of miR-216a and GDF15 in the Context of Different Immune Cell Contents

**Figure 3:**
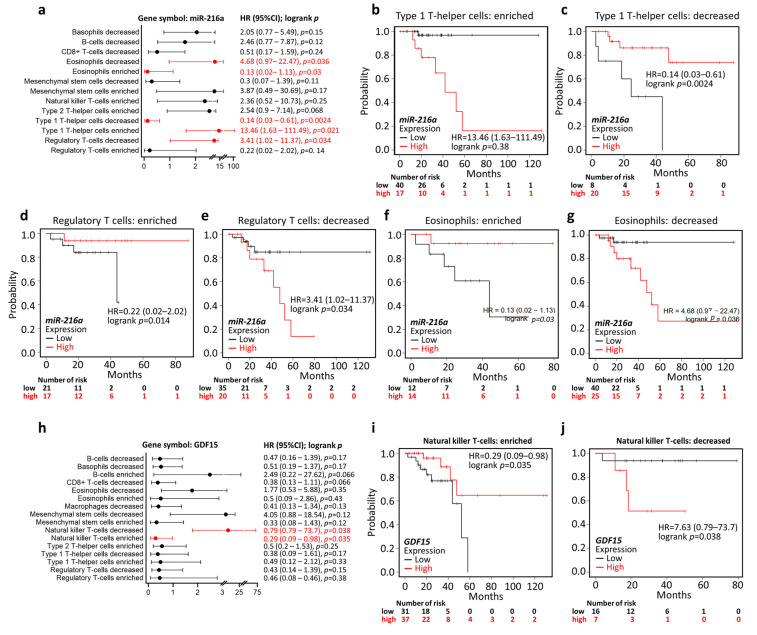
Immune cell contents altered the predictive significance of *miR-216a* and *GDF15*. (**a**) Forest plots summarizing the hazard ratio (HR), the 95% confidence interval (95% CI), and the logrank *p*-value of COAD miR-216a expression in the context of various immune cell contents. Kaplan–Meier analysis based on low/high miR-216a in the presence of enriched type 1 T-helper (Th1) cells (**b**), decreased Th1 cells (**c**), enriched regulatory T (Treg) cells (**d**), decreased Treg cells (**e**), enriched eosinophils (**f**), and decreased eosinophils (**g**). (**h**) Forest plots summarizing the HR, 95% CI, and logrank *p*-value of *GDF15* expression of COAD in the context of various immune cell contents. Kaplan–Meier analysis based on low/high *GDF15* expression in the presence of enriched natural killer T-cells (NKTs) (**i**) and decreased NKTs (**j**).

We further examined the Kaplan–Meier results in the context of different immune cell contents. High miR-216a-associated poor OS was reversed in the context of decreased type 1 T-helper (Th1) cells, enriched regulatory T (Treg) cells, and enriched eosinophils (Figure 3a–g). In addition, high *GDF15*-associated favorable OS was reversed in the presence of decreased nature killer T-cells (NKTs; Figure 3h–j). In light of the findings that the overall predictive value was reversed in the presence of certain immune cell content, we suggested that enriched Th1, enriched Treg, enriched eosinophils, and decreased NKTs may be counteracting factors on the tumor-regulatory role of miR-216a and *GDF15*.

### 3.4. Mutation Landscape of miR-216a and GDF15

**Figure 4:**
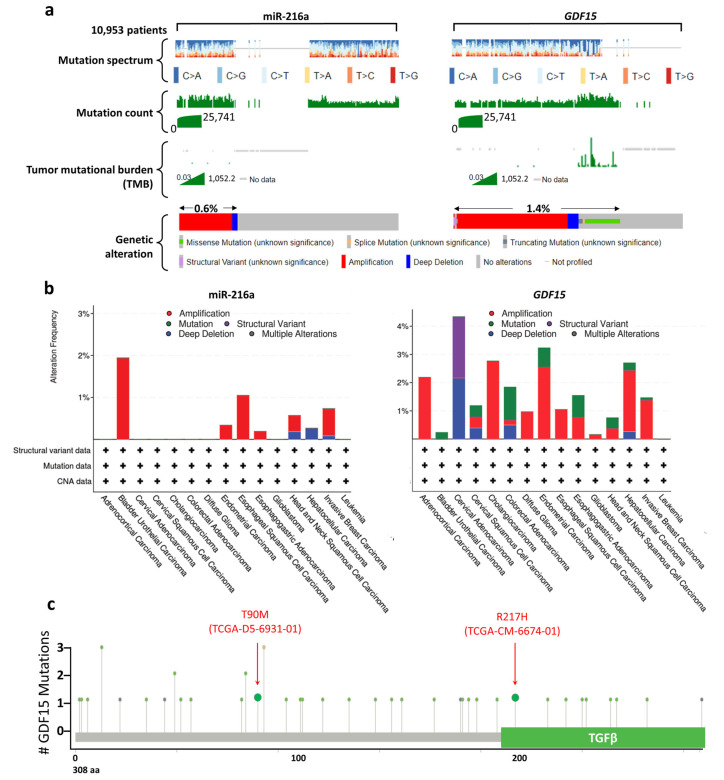
Mutation landscapes of miR-216a and *GDF15* based on cBioPortal. (**a**) The mutation spectrum, mutation counts, tumor mutational burden (TMB), and genetic alterations of miR-219a and *GDF15* across all cancer patients (*n* = 10,953). (**b**) The alteration frequency of miR-216a and GDF15 in various cancer types. (**c**) Schematic illustration of the mutation events on the GDF15 protein sequence.

We investigated the mutation landscape of miR-216a and *GDF15* in 10,953 patients. As shown in Figure 4a, the mutation spectra/counts of miR-216a and *GDF15* did not synchronize with the corresponding tumor mutational burden (TMB). The genetic alteration of miR-216a stood at 0.6%, mainly composed of amplification and deep deletion. The genetic alteration of *GDF15* was 1.4%, which primarily included amplification, deep deletion, missense mutation, truncated mutation, and structural variant. Interestingly, we noted that the TMB synchronized with the missense mutation and truncated mutation of *GDF15*. Nevertheless, no genetic alteration of miR-216a was noted in COAD, while that of *GDF15* stood at 1.85% of 594 cases (Figure 4b). The number and mutation distribution across *GDF15* is demonstrated in Figure 4c. Two missense mutations T90M (TCGA-D5-6391-01) and R217 (TCGA-CM-6674-01) were identified in COAD samples of TCGA PanCancer Atlas (Figure 4c).

### 3.5. Generalization Value of the miR-216a/GDF15 Axis in Pan-Cancer

**Figure 5:**
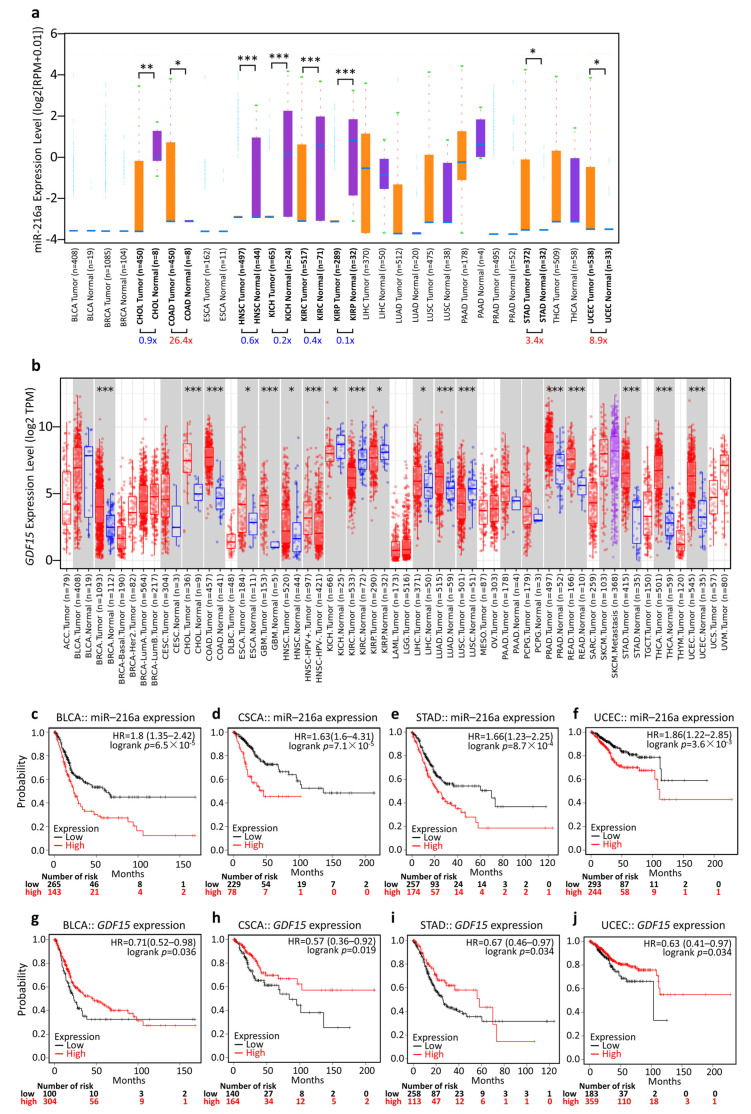
Generalization value of miR-216a/*GDF15* expression in pan-cancer diagnosis and prognosis. Expression profiles of miR-216a (**a**) and GDF15 (**b**) analyzed by ENCORI and Tumor Immunology Estimation Resource version (TIMER), respectively. Kaplan–Meier survival analysis representing the probability of the OS in various cancers based on miR-216a expression (**c**–**f**) and GDF15 expression (**g**–**j**). ACC, adrenocortical carcinoma; BLCA, bladder carcinoma; BRCA, breast cancer; CHOL, cholangiocarcinoma; CSCA, cervical squamous cell carcinoma; DLBC, lymphoid neoplasm diffuse large b-cell lymphoma; ESCA, esophageal squamous cell carcinoma, GBM, glioblastoma multiforme; HNSC, head–neck squamous cell carcinoma; KICH, kidney chromophobe; KIRC, kidney renal clear cell carcinoma; KIRP, kidney renal papillary cell carcinoma; LAML, acute myeloid leukemia; LIHC, liver hepatocellular carcinoma; LGG, brain lower grade glioma; LUAD, lung adenocarcinoma; LUSC, lung squamous cell carcinoma; MESO, mesothelioma; OV, ovarian cancer; PAAD, pancreatic ductal adenocarcinoma; PCPG, pheochromocytoma and paraganglioma; PRAD, prostate adenocarcinoma; SARC, sarcoma; STAD, stomach adenocarcinoma; TGCT, testicular germ cell tumor; THCA, thyroid carcinoma; THYM, thymoma; UCEC, uterine corpus endometrial carcinoma; UVM, uveal melanoma. * *p* < 0.05, ** *p* < 0.01, *** *p* < 0.001 tumor vs. normal.

To investigate whether miR-216a and GDF15 have a broad value, we performed a series of studies on miR-216a and GDF15 across all cancer types. We used ENCORI and TIMER analysis and revealed that miR-216a and GDF15 expression status varied in different cancers (Figure 5a,b). We noted that stomach adenocarcinoma (STAD) and uterine corpus endometrial carcinoma (UCEC) presented similar patterns to COAD, wherein tumors expressed high miR-216a expression levels along with high GDF15 expression compared to normal tissue (Figure 5a,b). The Kaplan–Meier analysis showed that high miR-216a expression had significant associations with short OS, while high GDF15 expression had significant associations with long OS in bladder carcinoma (BLCA), cervical squamous cell carcinoma (CSCA), stomach adenocarcinoma (STAD), and uterine corpus endometrial carcinoma (UCEC) (Figure 5c–j).

### 3.6. GDF15 Negatively Correlated with Immunoinhibitory Genes and Immune Infiltration

**Figure 6:**
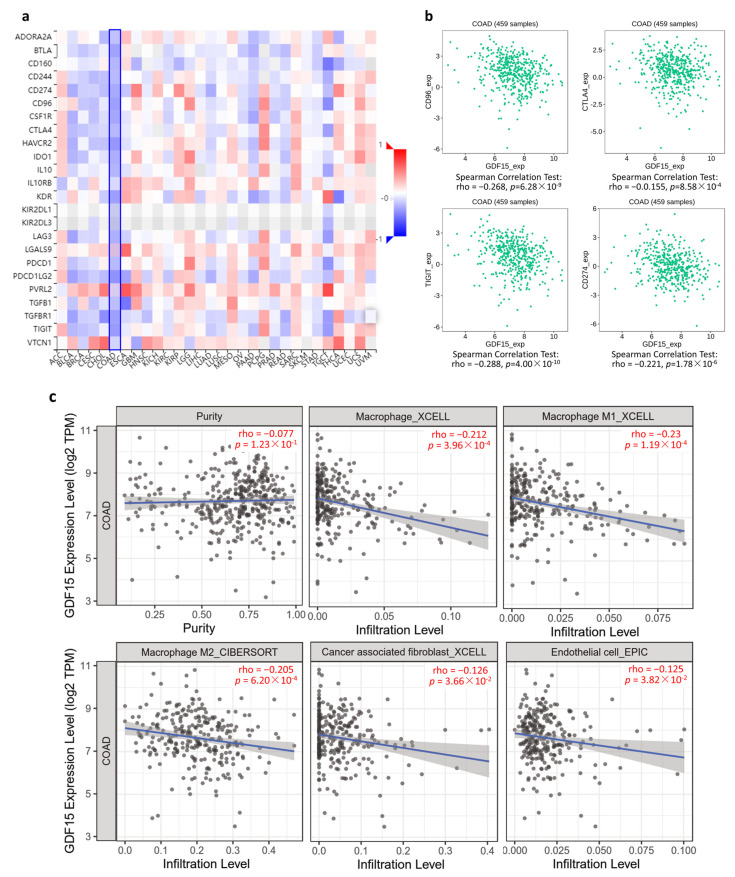
*GDF15* negatively correlated with immunoinhibitory genes and immune infiltration of macrophage, cancer associated fibroblast, and endothelial cells. (**a**) Correlation analysis between the expression of *GDF15* and 24 immunoinhibitory genes across human cancers based on TISIDB. (**b**) Spearman’s correlation between *GDF15* and CD96, TIGIT, CTLA4, and CD274 (PD-L1). (**c**) TIMER analysis of the purity-corrected Spearman’s correlation between the expression of *GDF15* and five immune cells in COAD.

The results of TISIDB demonstrated that *GDF15* negatively correlated with a majority of the immunoinhibitory genes in COAD tumor tissues (Figure 6a). In this regard, immunoinhibitors including cytotoxic T-lymphocyte-associated protein 4 (CTLA4), programmed death-ligand 1 (PD-L1, also known as CD274), CD96, and T cell immunoreceptor with Ig and ITIM domains (TIGIT) exhibited negative correlations with *GDF15* levels (Figure 6b). The TIMER2.0 analysis demonstrated that GDF15 had significant negative correlations with total macrophage, macrophage M1, macrophage M2, cancer-associated fibroblast, and endothelial cells (Figure 6c).

### 3.7. Co-Expression Network and Biological Functions of GDF15

**Figure 7:**
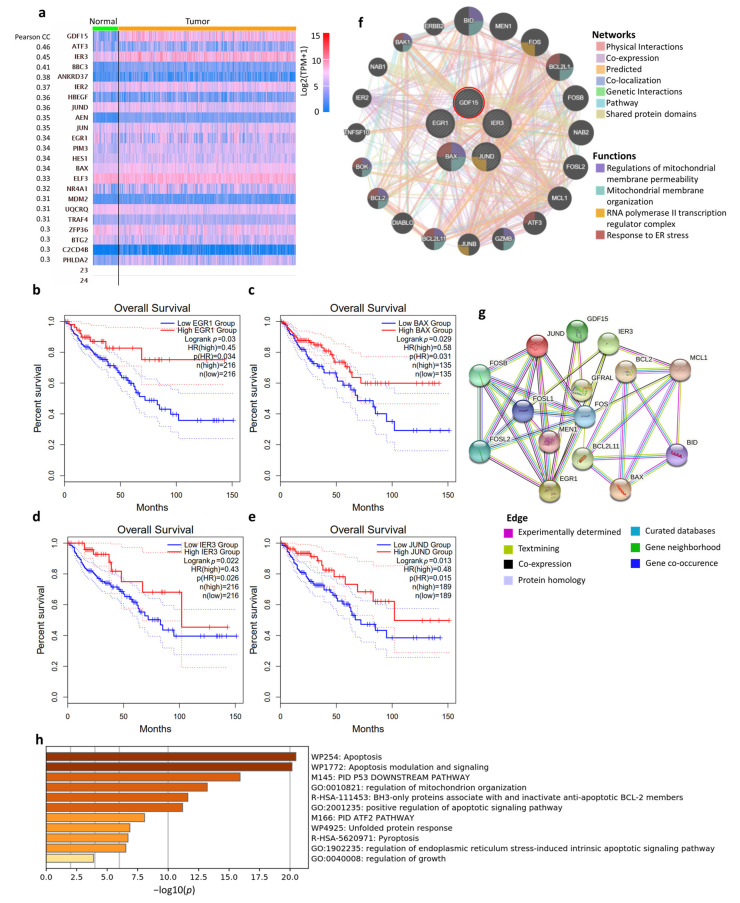
Potential mechanisms of GDF15 in COAD. Intergenic correlations, co-expression network, and the biological functions of *GDF15*. Genes were positively correlated with *GDF15* in COAD based on UALCAN analysis. (**a**) Pearson’s correlation coefficient of 22 genes with *GDF15* in normal and COAD tumor tissue. Kaplan–Meier analysis representing the probability of the OS in COAD based on the expressions of *EGR1* (**b**), *BAX* (**c**), *IER3* (**d**), and *JUND* (**e**). GeneMANIA analysis revealed the protein–protein interaction (PPI) among predicted functional partners after considering physical interaction, co-expression, prediction algorithm, co-localization, pathways, genetic interactions, and shared protein domains. (**f**) The enriched functions including the regulation of mitochondrial membrane permeability involved in apoptotic process, mitochondrial membrane organization, RNA polymerase II transcription regulator complex, and response to endoplasmic reticulum stress. (**g**) STRING analysis revealed mutual PPI, wherein the edges are verified by experimentally determined interactions, text mining, co-expression, protein homology, curated databases, gene neighborhood, and gene co-occurrence. (**h**) Heatmap of enriched ontology clusters based on Metascape analysis.

To gain molecular insights into the role of *GDF15* in COAD, we further used UALCAN analysis to acquire 22 genes positively correlated with *GDF15* in COAD tumor tissue (Figure 7a). Among these genes, high expressions of early growth response 1 (EGR1), BCL2-associated X protein (BAX), immediate early response 3 (IER3), and JunD Proto-Oncogene (JUND) were identified to have associations with longer OS (Figure 7b–e). The PPI analysis revealed that the co-expression networks mainly participate in mitochondrial organization, apoptosis process, transcriptional activity, and endoplasmic reticulum (ER) stress (Figure 7f,g). After constructing this network, we applied the Metascape algorithm that processes enrichment analysis integrating gene ontology sources, including the GO biological process, the KEGG pathway, Reactome gene sets, and canonical pathways. As shown in Figure 7h, the top 20 clusters of the enriched sets were identified. As such, these genes were enriched in the molecular categories implicated in mitochondrial organization, apoptosis signaling, unfolded protein response, pyroptosis (a type of immunogenic cell death), and ER stress response.

### 3.8. Gemcitabine as a Therapeutic Option in the Context of Low GDF15

**Figure 8:**
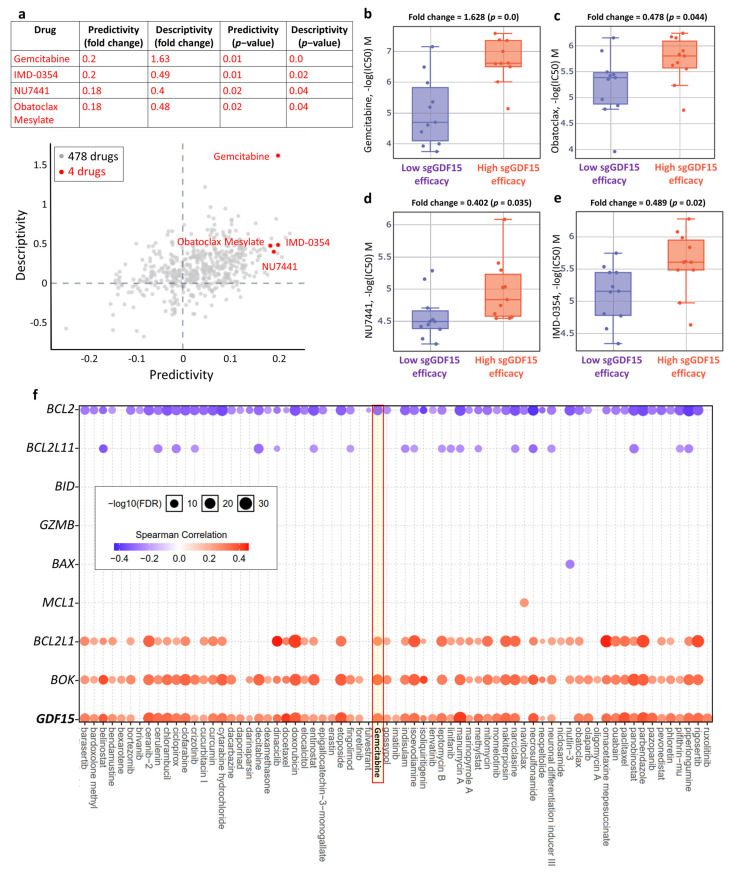
Gemcitabine as a therapeutic option in the context of low *GDF15*. Q-omics analysis was used to analyze cross-association scores regarding predictivity and descriptivity for the identification of potent drugs acting on large intestine cells based on *GDF15*. (**a**) Predictivity, fold change of single guide GDF15 (sgGDF15) efficacy (sgRNA efficiency of *GDF15* knockout) between cells of high and low responses of the target drug. Descriptivity, fold change of target drug response between samples of high and low sgGDF15 efficacy. Red dots represent hits with a predictivity *p*-value of <0.05 and a descriptivity *p*-value of <0.05. Boxplots of −log(half maximal inhibitory concentration (IC_50_)) M of Gemcitabine (**b**), obatoclax mesylate (**c**), NU7441 (**d**), and IMD-0354 (**e**) in large intestine cells with low (group 1) and high (group) sgRNA efficacy of *GDF15*. (**f**) List of Spearman’s correlation array between drug IC_50_ and gene expression, including *GDF15*, *BOK*, *BCL2L1*, *MCL1*, *BAX*, *GZMB*, *BID*, *BCL2L11*, and *BCL2*. Note that a positive correlation means that the gene’s low expression indicates drug sensitivity, and vice versa.

To explore potential pharmaceutical approaches that can effectively target COAD, we utilized the GDSC repository in Q-omics analysis to identify potent drugs that may act to activate *GDF15*. We conducted cross-associations between drug response and single guide RNA (sgRNA)-mediated *GDF15* knockdown based on the CRISPR approach in large intestine cells. As shown in Figure 8a, four out of 478 drugs were identified, including Gemcitabine, NU7441, IMD-0354, and obatoclax mesylate. Moreover, large intestine cells with high sgRNA-GDF15 efficiency exhibited higher log(IC_50_) values in response to Gemcitabine (Figure 8b), obatoclax mesylate (Figure 8c), NU7441 (Figure 8d), and IMD-0354 (Figure 8e). Among the four drugs, Gemcitabine showed the highest predictivity and descriptibility, as well as the highest fold change in the scenario of high *sgGDF15* efficiency, indicating that Gemcitabine exerted the most significant suppressive effect in the context of *GDF15* deficiency. In addition, we examined the relationship between *GDF15* expression and drug sensitivity using the CTRP IC_50_ drug data repository. As shown in Figure 8f, Gemcitabine showed a positive correlation with *GDF15* expression, indicating that low *GDF15* expression was sensitive to Gemcitabine. Collectively, these results suggested that Gemcitabine can serve as a therapeutic option when *GDF15* levels are low.

**Figure 9:**
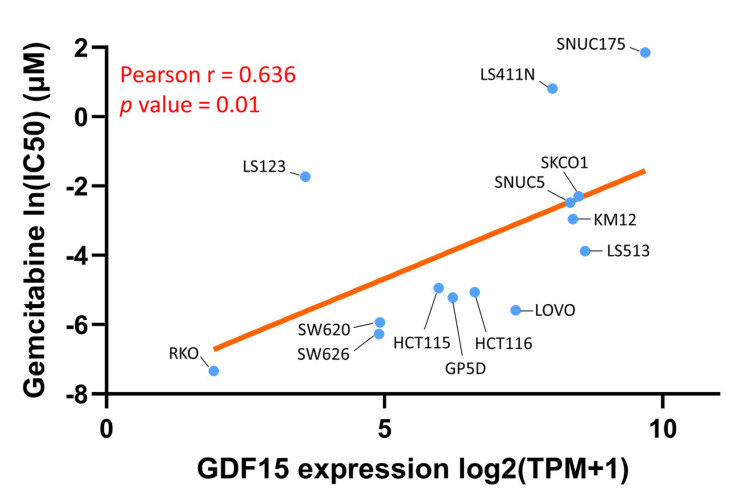
The relationship between *GDF15* expression levels and Gemcitabine sensitivity in 14 COAD cell lines. The Pearson’s correlation of *GDF15* expression levels were expressed as log2 of transcripts per million (TPM), and Gemcitabine sensitivity was expressed as the nature log of IC_50_.

We then employed CCLE analysis to further verify the effect of *GDF15* expression levels on Gemcitabine sensitivity in 14 COAD cell lines. As shown in Figure 9, the Gemcitabine sensitivity featured a positive correlation with *GDF15* expression (Pearson r = 0.636, *p*-value = 0.01), supporting the notion that Gemcitabine serves as a therapeutic option in the context of low *GDF15* expression.

## 4. Discussion

**Figure 10:**
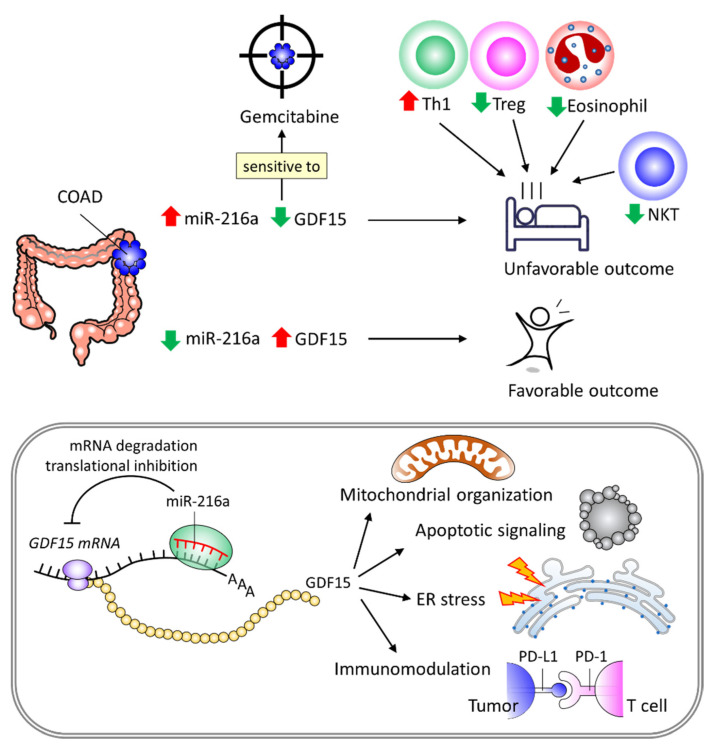
Proposed model depicting the significance of the miR-216a/*GDF15* axis in diagnosis, prognosis, tumor immunity, and precision treatment with Gemcitabine in COAD and its correlation for mitochondrial organization, apoptotic signaling, endoplasmic reticulum (ER) stress, and immunomodulation.

The biological role and clinical implications of miR-216a and *GDF15* in COAD remain unclear. In this study, we employed a variety of databases to reveal elevated miR-216a and *GDF15* levels as diagnostic biomarkers and as independent risk predictors for favorable and unfavorable outcomes, respectively. We demonstrated that the miR-216a/*GDF15* axis may account for the underlying molecular basis of COAD progression, offering an opportunity for the development of anti-miR-216a and *GDF15*-mimic approaches as therapeutic strategies. In addition, we revealed that tumor immune cell contents, such as enriched Th1, enriched Treg, enriched eosinophils, and decreased NKT, may play counteracting roles in the tumor-regulatory activity of the miR-216a/*GDF15* axis. Furthermore, we found that the diagnostic and prognostic value of miR-216a and *GDF15* in COAD can be extrapolated to other cancer types, including BLCA, CSCA, STAD, and UCEC. The favorable survival time associated with high *GDF15* expression levels may be due to the suppression of immunoinhibitory genes and reduced immune infiltration of macrophage, cancer-associated fibroblast and endothelial cells, as well as perturbed mitochondrial organization, apoptosis signaling, ER stress, and immunomodulation. Importantly, our investigation indicated that Gemcitabine can act as a therapeutic option when present with low *GDF15* expression levels, offering a promising precision treatment strategy. A proposed model is summarized in Figure 10.

As an independent predictor, *GDF15* was reported to be overexpressed in many tumors, including colon, prostate, and breast [38,39,40,41]. In addition, Danta et al. demonstrated that baseline GDF15 serum levels increase in subjects with polyps compared to those with no polyps and that the levels decreased in post-polypectomy subjects compared to in pre-polypectomy ones, indicating the significance of GDF15 as a predictive factor in colonic neoplasia [42]. Our study revealed that the high expression of *GDF15* had associations with COAD tissue, supporting its value as a diagnostic/prognostic marker. As a mitokine in response to mitochondrial stress, the role of *GDF15* in mediating stress resistance to promote cellular longevity through modulating mitochondrial homeostasis is gradually emerging [43]. Kang et al. reported that the upregulation of *GDF15* levels is induced by mitochondrial dysfunction. The knockdown of *GDF15* impedes metastatic behaviors, indicating the tumor-promoting effect of *GDF15* in thyroid cancer [44]. Furthermore, *GDF15′*s tumor promoting role in COAD has been noted in several reports [9,10,11]. For instance, secreted GDF15 of fibroblasts was identified to be involved in fostering a senescence-associated tumor microenvironment that promotes proliferation and invasion of COAD cells [45]. On the other hand, some studies demonstrated the role of *GDF15* in mediating the anti-tumoral activity of pharmacological treatments. Shin et al., in this regard, reported that *GDF15* is required for 2’-hydroxyflavanone-induced apoptosis in the HCT116 colon cancer cell line [46]. Guo et al. have indicated that *GDF15* acts as a tumor suppressor of COAD in the context of traditional Chinese medicine treatment [12]. In this study, we revealed that an elevated *GDF15* expression level is an independent predictor for longer survival time. However, the question of whether *GDF15* exerts anti-COAD effects requires further in-depth study.

Wei et al. demonstrated that COAD tumor presents higher levels of miR-216a than normal issue and that the high expression of miR-216a serves as one prognostic factor for shorter OS time [47]. This is in tandem with the results of our study. Nevertheless, one limitation of our result with respect to miR-216a expression levels was the small sample size, which may lead to statistical Type II error. Further study with a larger sample size is warranted. Whether miR-216a acts to promote or suppress COAD remains a debatable issue. In this study, the TCGA dataset shows that elevated miR-216a levels is an independent risk factor for COAD patients, suggesting that miR-216a may be a positive factor for COAD progression. This notion is in line with a study reported by Zeng et al., which revealed that downregulated miR-216a acts toward COAD suppression [22]. In addition, the fact that miR-216a binds to *GDF15* 3′UTR and that there exists a reverse prognostic outcome between the two markers suggests that the activity of the miR-216a/*GDF15* axis may have associations in controlling COAD progression. However, a major caveat of this study lies in the majority use of database analysis that is in need of further molecular verification through an investigation to clarify the exact mechanisms in a COAD experimental model. While further study in this regard is warranted, our study reveals novel insights into the possible role of the miR-216a/*GDF15* axis in COAD.

One critical factor impacting on CRC development is gut microbiota, which can act to promote oncogenic transformation and alter tumor-associated inflammation by perturbing redox status and the activity of immune cells [48]. Wang et al. demonstrated that *Enterococcus faecalis* infection in macrophage promotes superoxide production, leading to DNA damage in epithelial cells via a bystander effect [49,50]. Mangerich et al. showed that *Helicobacter hepaticus* plays a role in the accumulation of macrophage and neutrophil in the colon and acts to promote nitric oxide (NO) production. These effects have been observed to occur concomitantly with gut inflammation and colon cancer development [51]. In this study, we demonstrated that *GDF15* expression inversely correlated with tumor-associated macrophage. That raises a possibility that macrophage may be a common target of *GDF15* and gut microbiota in the context of colonic tumorigenesis and that *GDF15* may interplay with gut microbiota to regulate immune response. Nevertheless, more investigations are needed to clarify the exact mechanisms.

Gemcitabine (brand name Gemzar) is an FDA-approved drug for pancreatic, lung, and metastatic breast cancer, reaching phase 2 clinical trials (NCT00220155 and NCT00007943). Zheng et al. reported on the anti-tumoral effect of Gemcitabine in a mouse CT26 COAD model [52]. Several clinical studies have revealed that Gemcitabine is clinically active and well-tolerated when combined with the chemotherapeutic agent fluoropyrimidine [53]. Saif et al. have also suggested that the combination of Gemcitabine and capecitabine is an effective option for the treatment of CRC patients in advanced stages [54]. However, studies into Gemcitabine prescription adhering to the principles of precision treatment are lacking. It is noteworthy that the present study reveals Gemcitabine as a potential therapeutic strategy for COAD in the presence of low *GDF15* expression levels. While more studies are warranted, this finding may support the development thesis of a precision treatment for COAD based on *GDF15* expression levels.

## 5. Conclusions

This study indicated the miR-216a/*GDF15* axis as a diagnostic/prognostic panel for COAD. We further identified Th1, Treg, eosinophils, and NKTs as counteracting factors and indicated potential relationships underlying immunomodulation, mitochondrial organization, apoptotic signaling, and ER stress. Our study data also revealed that Gemcitabine may be a potential drug for the development of a treatment strategy when combined with *GDF15* targeting.

## Data Availability

Data are contained within the article.

## Supplementary Materials

The following are available online at https://www.mdpi.com/article/10.3390/ijms222413636/s1.
