# Supplementary material for: Multi-Omics Reveal the Immunological Role and the Theragnostic Value of miR-216a/GDF15 Axis in Human Colon Adenocarcinoma
Source: Int J Mol Sci. 2021 Dec 20;22(24):13636. doi: 10.3390/ijms222413636 (PMC8703770; doi:10.3390/ijms222413636)
Supplement: Supplementary file 1 [file ijms-22-13636-s001.zip › ijms-1480430-supplementary.pdf]

Supplementary Materials

Multi-omics reveal the Immunological Role and the Theragnostic Value of miR-216a/GDF15 axis in Human Colon Adenocarcinoma

Chun-Bin Tung, Chia-Ying Li *, Hung-Yu Lin *

Filtering criteria for multi-omic analysis

For UALCAN analysis (http://ualcan.path.uab.edu/) (accessed on October 25, 2021), TCGA Gene analysis was employed to retrieve TCGA dataset of Colon adenocarcinoma. The "Expression" link was selected to display gene expression profile and the statistical significance between different sample types. For the display of the positively correlated genes, we used "Correlated genes" link to demonstrate the heatmeap and the Pearson correlation coefficient. For ENCORI analysis (https://starbase.sysu.edu.cn/index.php) (accessed on October 25, 2021), the gene expression profile and miRNA-mRNA correlation were examined using "Gene Differential Expression" and "miRNA-Target" panel, respectively. In addition, ENCORI was used to conduct pan-cancer analysis of gene expression profile. For representative IHC imaging on The Human Protein Atlas (https://www.proteinatlas.org/) (accessed on October 25, 2021), normal colon and matched tumor tissue probed with the same antibody were compared. For TargetScan analysis, at least 7-mer sites of the mRNA 3'UTR matching with the miRNA was analyzed. For survival analysis in Kaplan-Meier Plotter (https://kmplot.com/analysis/) (accessed on October 25, 2021), patient groups were divided by "Auto select best cutoff", which automatically computes all possible cutoff values between the lower and upper quantile and selects the best performing threshold as a cutoff. For GEPIA-based survival analysis (http://gepia2.cancer-pku.cn/#index) (accessed on October 25, 2021), the best performing cutoff was screened and applied to divide patient groups. For cBioPortal analysis, only mutation events identified in COAD samples (TCGA-COAD) were highlighted. For TISIDB analysis (http://cis.hku.hk/TISIDB/) (accessed on October 25, 2021), the default was applied to demonstrate the heatmap of Spearman correlations rho value between the gene of interest and the immunoinhibitors across cancers. For TIMER analysis (http://timer.cistrome.org/) (accessed on October 25, 2021), the "Purity Adjustment" option was considered to display the spearman's rho value between the gene of interest and immune infiltration level in tumor samples. For GeneMANIA analysis of PPI (https://genemania.org/) (accessed on October 25, 2021), all network options such as physical interactions, co-expression, predicted, co-localization, genetic interactions, pathway, and shared protein domains were selected. The FDR < 0.05 is the inclusion criteria for the enrichment of gene functions. For STRING analysis of PPI (https://string-db.org/)

(accessed on October 25, 2021), the minimum required interaction score was set at 0.7 (high confidence). For Metascape analysis (https://metascape.org/gp/index.html#/main/step1) (accessed on October 25, 2021), all statistically enriched terms, accumulative hypergeometric p-values and enrichment factors were calculated and used for filtering. The -log10(P) > 2.5 was set as a minimal requirement. For Q-omics analysis, the "Cross-association" option was used to determine the association between CRISPR-screened genes and drug sensitivity, wherein the "Sample" option was limited to "Large Intestine" and data grouping was set at median cutoff. For GSCALite platform (http://bioinfo.life.hust.edu.cn/web/GSCALite/) (accessed on October 25, 2021), the "Screen Cancer" option was limited to "TCGA-COAD" and the "Select Analysis" option included Drug Sensitivity. The correlation between gene expression and drug sensitivity (IC50) was performed by Spearman correlation analysis, which displayed the rho with -log(FDR) greater than 10. For CCLE analysis (https://sites.broadinstitute.org/ccle/tools) (accessed on November 26, 2021) for gene-drug correlation, *GDF15* expression was retrieved from Expression 21Q4 Public while Gemcitabine data was accessed from Drug Sensitivity IC50 (GDSC1 and GDSC2).

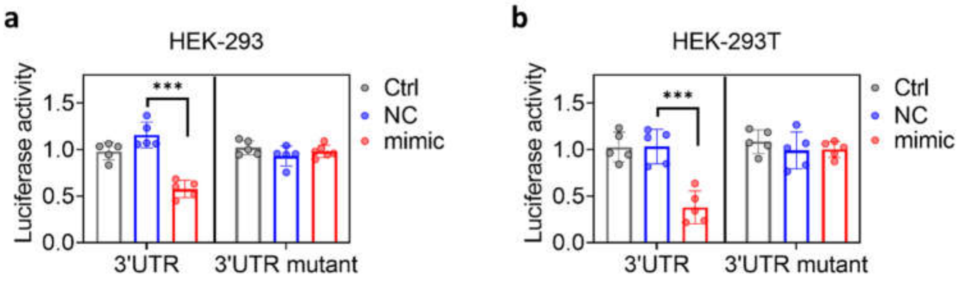

Figure S1. miR-216a-3p mimic-mediated suppression of luciferase signal in HEK-293 (a) and HEK-293T (b) cells (n=5). Ctrl, untreated control. control. NC, negative sequence control. ***p<0.001 between indicated groups.
